# Multi-Scale Biomechanical Remodeling in Aging and Genetic Mutant Murine Mitral Valve Leaflets: Insights into Marfan Syndrome

**DOI:** 10.1371/journal.pone.0044639

**Published:** 2012-09-11

**Authors:** Russell A. Gould, Ravi Sinha, Hamza Aziz, Rosanne Rouf, Harry C. Dietz, Daniel P. Judge, Jonathan Butcher

**Affiliations:** 1 Department of Biomedical Engineering, Cornell University, Ithaca, New York, United States of America; 2 Johns Hopkins University School of Medicine, Baltimore, Maryland, United States of America; 3 Howard Hughes Medical Institute, Bethesda, Maryland, United States of America; University of Arizona, United States of America

## Abstract

Mitral valve degeneration is a key component of the pathophysiology of Marfan syndrome. The biomechanical consequences of aging and genetic mutation in mitral valves are poorly understood because of limited tools to study this in mouse models. Our aim was to determine the global biomechanical and local cell-matrix deformation relationships in the aging and Marfan related *Fbn1* mutated murine mitral valve. To conduct this investigation, a novel stretching apparatus and gripping method was implemented to directly quantify both global tissue biomechanics and local cellular deformation and matrix fiber realignment in murine mitral valves. Excised mitral valve leaflets from wild-type and *Fbn1* mutant mice from 2 weeks to 10 months in age were tested in circumferential orientation under continuous laser optical imaging. Mouse mitral valves stiffen with age, correlating with increases in collagen fraction and matrix fiber alignment. *Fbn1* mutation resulted in significantly more compliant valves (modulus 1.34±0.12 vs. 2.51±0.31 MPa, respectively, P<.01) at 4 months, corresponding with an increase in proportion of GAGs and decrease in elastin fraction. Local cellular deformation and fiber alignment change linearly with global tissue stretch, and these slopes become more extreme with aging. In comparison, *Fbn1* mutated valves have decoupled cellular deformation and fiber alignment with tissue stretch. Taken together, quantitative understanding of multi-scale murine planar tissue biomechanics is essential for establishing consequences of aging and genetic mutations. Decoupling of local cell-matrix deformation kinematics with global tissue stretch may be an important mechanism of normal and pathological biomechanical remodeling in valves.

## Introduction

Marfan syndrome (MFS) is an autosomal-dominant systemic disorder of connective tissue, with an estimated prevalence of 1 in 5,000 individuals [1]. It is associated with mutations in *Fbn1*, encoding fibrillin-1, the principal component of extracellular microfibrils, leading to severe cardiovascular, ocular, and skeletal defects [2]. Of these consequences, mitral valve disease is one of the leading indications for surgery and causes of death in young children with MFS. Furthermore, mitral valve prolapse is widely prevalent, affecting 2.4% of individuals in a community-based survey [3]. Currently, no medical therapies exist to prevent valve disease in predisposed individuals [4]. Despite its significant public health and clinical burden, very little is known about the biomechanical remodeling of mitral valves with age or genetic mutations, which lie at the core of its pathophysiology.

Mitral valves (MV) are fibrous leaflets whose primary function is to maintain unidirectional blood inflow into the left ventricle with each heartbeat [5]. Resident valve interstitial cells (VIC) repair and remodel the tissue to form a complex biomechanical structure capable of withstanding forces exerted by the surrounding blood and muscle walls [6]. MV tissue biomechanics is dictated in large part by local microstructure, which is comprised of stratified collagen and elastin dispersed with proteoglycans [7]. Myxomatous MV leaflets have increased production of collagens, particularly type III collagen, and glycosaminoglycans (GAGs) [8]. Some postulate that this may be the result of a leaflet response to repeated mechanical stress leading to MV dysfunction [9]. Sherratt et al. suggested that microfibrils, predominantly composed of fibrillin-1, act as stiff reinforcing filaments in elastic fibers, possibly limiting the extension of elastin and protecting these fibers from hemodynamic damage [10]. Breakdown of these microfibrils may also affect adjacent cell attachment, as an RGD sequence of fibrillin-1 has been reported to support cellular adhesion in vitro via integrin α_v_β_3_ [11], [12]. Still, the global and local biomechanical consequences of human fibrillin-1 mutations in the mitral valve have not been fully elucidated.

Mouse models are indispensible for understanding functional consequences of fibrillin-1 mutations. Ng et al. found that MV leaflets from mice with a heterozygous cysteine-substitution mutation in fibrillin-1 exhibit postnatally acquired alterations in architecture that correlate both temporally and spatially with increased cell proliferation, decreased apoptosis, and excess TGF-β activation and signaling [13]. Mice homozygous for a hypomorphic *Fbn1* allele (mgR) die between 3 and 6 months of age from aortic dissections. This tissue mechanical failure is preceded by a series of secondary events that are spatially coincident including progressive elastic fiber degradation and disarray and aortic wall thickening due to excessive deposition of matrix elements including collagen and proteoglycans. These findings suggest that cell mediated matrix remodeling leads to gross tissue biomechanical changes. While molecular processes are easily studied in mice, no experimental devices exist for quantifying the biomechanical characteristics of these micro-scale planar soft tissues.

In this study, we implement a novel method to quantify global biomechanical and local cell-matrix relationships in small-scale planar tissues. By stretching mouse mitral valves under confocal microscopy, tissue strain was quantified concurrently with underlying cellular deformation and fiber alignment. We found that local cell and matrix kinematics are decoupled to different degrees correlating with magnitude of global valve tissue stretch. This decoupling was correlated with transitions in the proportions of collagen and GAGs, whether with age or *Fbn1* mutation.

## Materials and Methods

### Device Fabrication and Biomechanical Testing

**Figure 1:**
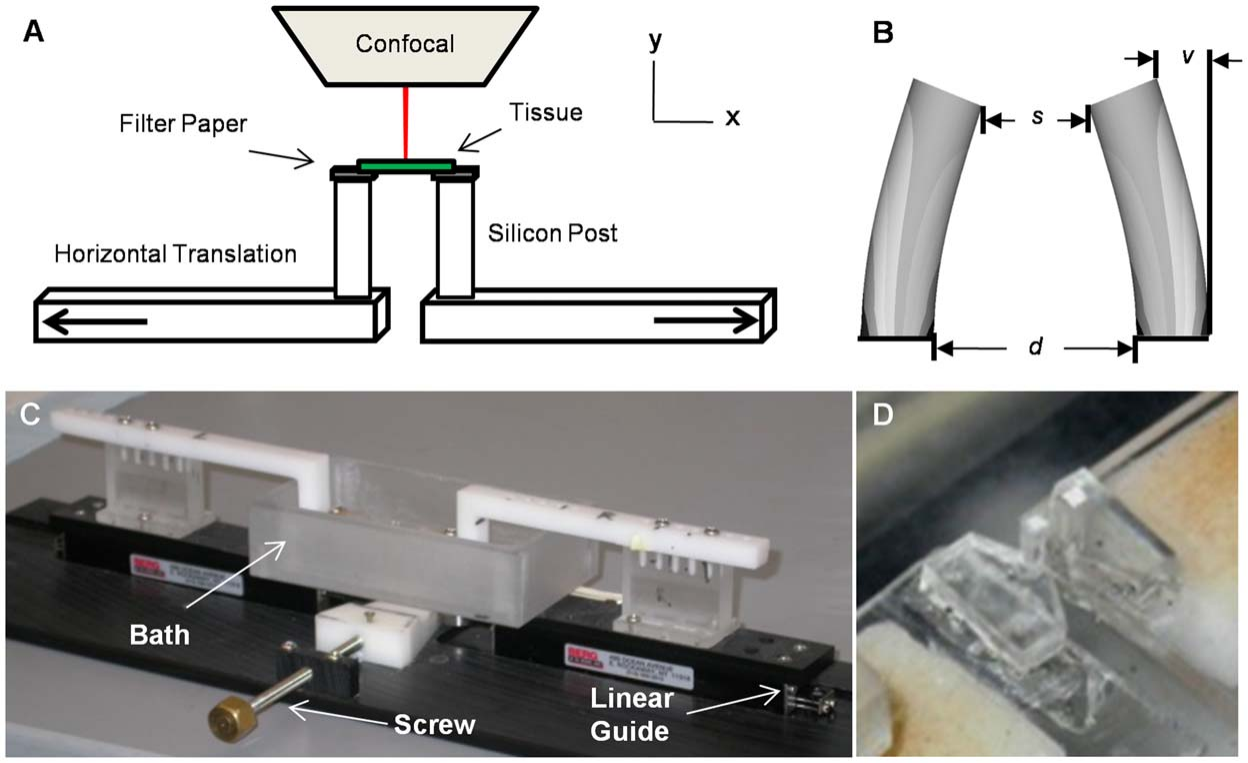
Meso-scale uniaxial tensile device. (A) Experimental stage controls the opposite co-linear translation of two cylindrical elastomeric beams. Tissue is mounted on the top surface of the posts using filter paper. The assembly is fully submerged in a buffered saline bath, and able to be imaged continuously under an upright confocal system. (B) Silicon post defection (v), post separation (s), and horizontal translation (d) were used for the biomechanical analysis. (C) Fabricated device implements a screw-driven wedge design for separation of the two linear rail guides at micro-meter resolution. (D) Detailed view of the silicon-post setup located inside the water bath. Distance between the posts is approximately 500 um.

We designed and fabricated a device that miniaturized a classical uniaxial strip test (**Fig. 1A**). Using classical Euler cantilever beam deflection mechanics, elastomeric posts (Polydimethylsiloxane, Sylgard184) were used to measure micro-millinewton forces. Bending stiffness was calculated analytically with the measured deflection (**Fig. 1B**), and measured force verified independently via mass balance (**Fig. S1**) as previously described [14]. Cylindrical posts with a diameter of 2 mm and axial length of 6 mm were used for mitral valve testing. Once calibrated, the silicon posts were mounted on top of two L-shaped plastic beams that traveled on linear rail guides. A screw-driven wedge enabled equal and opposite co-linear displacement of the L-beams, and thus the cantilever posts, micron resolution (**Fig. 1C–D**).

### Mitral Valve Isolation and Biomechanical Testing

Wildtype (*Fbn1*+/+) and heterozygous (^C1039G/+^ *Fbn1*) knock-in mice between the ages of 2 weeks and 10 months were used in this study, as that reflects a broad range of murine valve growth and development [15]. Details of the human *Fbn1* knock-in mutation (C1039G/+) have been previously described [16]. The anterior mitral valve leaflet was dissected and the presence and architecture of cells and extracellular matrix (ECM) were visualized using fluorescent vital dyes (5 µM CellTrackerRed CMPTX and 10 µM 5-DTAF, respectively, both from Invitrogen). During the quasi-static tensile test, macroscopic tissue views of the valve were taken at 10× magnification, while microscopic z-stacks of cells and fibers at 40×, both using confocal microscopy. All mouse work was conducted with Institutional Care and Use Committees (IACUC) approval.

### Determination of Local Biomechanics

Biomechanical parameters were quantified through image analysis performed using ImageJ (NIH). Global tissue deformation was measured via tip-to-tip post displacement, while local tissue deformation at each incremental stretch were measured at the midline zone by tracking virtual fiducial markers using confocal microscopy as previously described [17]. The effective tissue modulus was determined using the two material coefficients (α,β) as previously described [18]. Local matrix fiber alignment and cell deformation was captured at each stretch interval using 40× confocal z-stacks (**Fig. S2**). Cell shape changes were traced manually, tracked over subsequent images, and quantified as a cell circularity index (CI = 4*Pi*(Area/Perimeter^2^)) as manually traced in ImageJ. Fiber alignment along the axis of stretch was quantified as an alignment index (FAI) custom algorithm through MATLAB as previously described [19].

### Extracellular Matrix Composition

Additional hearts were excised and fixed in 10% buffered formalin overnight, arranged in 1.5% agar prior to paraffin embedding, and slides were prepared using 7 µm sections. Sections of paraffin-embedded tissue were prepared and stained with either Movat Pentachrome, Masson’s Trichrome, or Verhoff-van Gieson stain (VVG). Colors representing different matrix constituents were separated in NIH ImageJ using an RGB or CMYK channel splitter and converted to grayscale images. These areas were then thresholded, quantified, and normalized against the area of the entire valve leaflet to determine relative fractional composition as previously described [20] (**Fig. S3**).

### Statistics

At least 6 independent valve specimens per time point and treatment condition were used during mechanical testing and samples from at least 3 independent valves were used for histochemical/morphologic analysis. All results are expressed as mean ± standard deviation, unless otherwise stated. One-way ANOVA with Tukey’s modified post-hoc tests were used to compare differences between means and data was transformed when necessary to obtain equal sample variances. For *Fbn1*-mutant mice, Student’s t-tests compared single time point across treatment conditions. P<0.05 denoted statistical significance. See extended methods (**Materials S1**).

## Results

### Temporal Biomechanical Analysis of Wild-type C57BL/6J Mitral Valves

**Figure 2:**
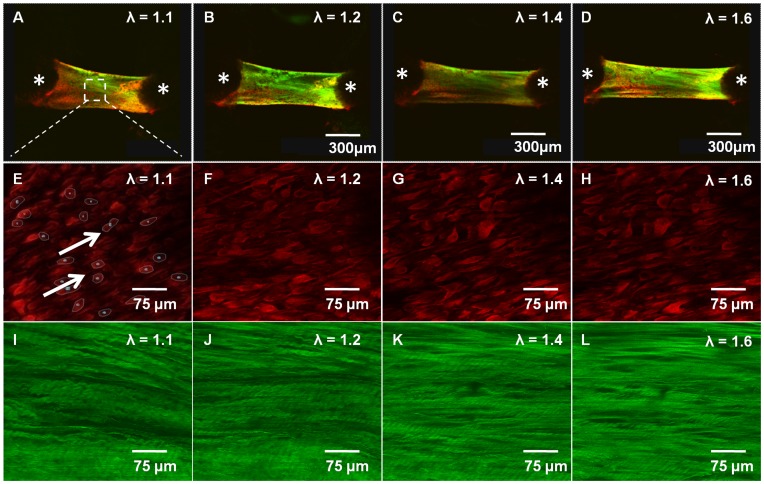
Macro and micro-scale tissue analysis. (A–D) Macro scale valve deformation at varying stretch ratios (λ) viewed at 10× under confocal microscopy (Green: 5-DTAF stained matrix fibers, Red: vital dye labeled cell bodies). Stars denote centroid location of the cantilever posts. (E–H) Micro-scale cellular deformation of the same group of cells viewed during the same test at 40X under confocal microscopy. Arrows denote tracked morphology of individual cells. (I–L) Micro-scale fiber alignment from the same region viewed during the same test at 40× under confocal microscopy. Fiber un-crimping and alignment is clearly visible as stretch progresses left to right.

**Figure 3:**
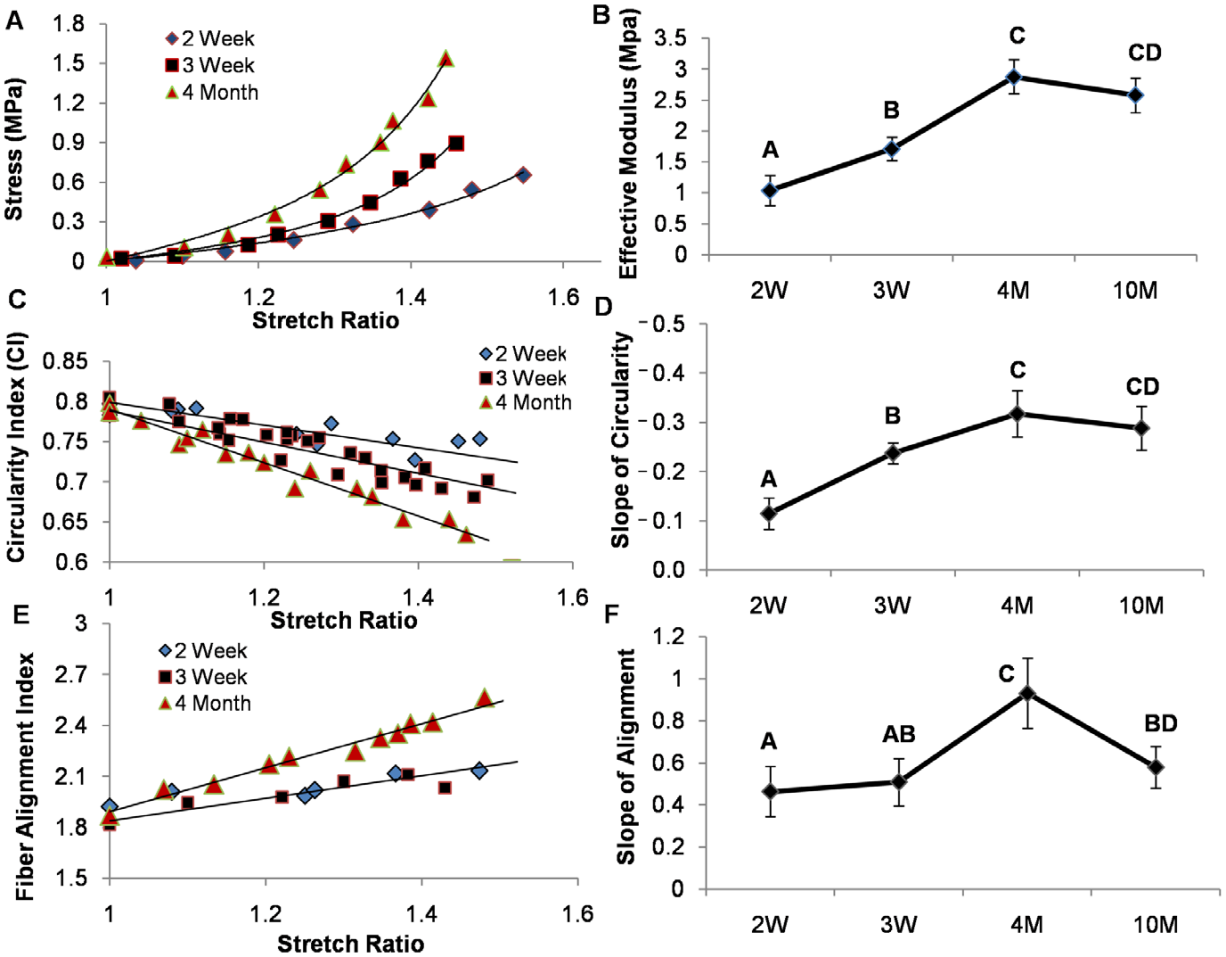
Temporal biomechanical analysis of C57/B6J mitral valves. (A) Representative stress-strain curves of mitral valves loaded in the circumferential direction (not to failure). (B) Stress-strain data were fit to an exponential Fung model, from which coefficients were used to determine effective modulus. (C) Representative circularity index curves as defined by the ratio of 4*Pi*(Area/Perimeter^2^). (D) Circularity-index curves were modeled as a linear fit and the negative slope was used for comparison. (E) Representative fiber-alignment curves were defined by the Fourier-Transform of collagen alignment and summed within ten degrees of the image horizontal, which was parallel to the tissue and loading direction. (F) Fiber-alignment data were fit to a linear model, the negative slope of which was used for comparison. Error bars show ±SD, n ≥6 for each condition. Groups that do not share letters are significantly different from each other according to a one-way ANOVA with Tukey’s post hoc (p≤0.05).

We tested circumferentially oriented mitral valve leaflet specimens from 2 week-, 3 week-, 4 month-, and 10 month-old C57BL/6J mice, an interval during which significant physiological and morphological changes (valve thickness, mitral annulus, and blood pressure) have been observed [15]. As our objective was to compare local and global tissue deformations, tissues were stretched to a maximum of λ = 1.6, which was sufficient to induce large cell shape changes but not cause fiber disruption or fracture. Macroscopically, the planar valves deformed as a typical uniaxial strip, with necking in the central region (**Fig. 2A–D**). Microscopically, resident cells steadily changed shape from round to elliptical with stretch (**Fig. 2E–H**), while matrix fibers progressively straightened and aligned to the loading direction (**Fig. 2I–L**). Biomechanically, anterior leaflets at each age exhibited nonlinear elastic material responses (**Fig. 3A**). Valve leaflets significantly increased in stiffness with maturation (effective modulus of 1.04±0.24 MPa at 2 weeks to 2.88±0.27 MPa at 4 months, P<0.05), after which stiffness was maintained for the next 6 months of life (2.58±0.28 MPa at 10 months, not different from 4 months) (**Fig. 3B**). Quantification of local cell deformation revealed a linear reduction in circularity index (CI) with stretch (**Fig. 3C**). The slope of CI became increasingly negative over maturation (–0.11±0.03 at 2 weeks to –0.32±0.05 at 4 months, P<0.05), but leveled off at 10 months (–0.28±0.4) (**Fig. 3D**). From these data, we were able to visualize a correlation between the effective tissue modulus and slope of CI, which suggests that cellular deformation increases with both stretch and tissue stiffness. Similarly, 5-DTAF labeled extracellular matrix fibers aligned linearly with stretch, and the degree of alignment significantly increased with tissue maturation (**Fig. 3E**, **S4**). The slope of fiber alignment (FA) index increased from 0.46±0.12 at 2 weeks to 0.93±0.17 at 4 months (P<0.05). In contrast to cell shape, FA slope decreased significantly at 10 months (0.58±0.09), which suggests that older valves have a reduced capacity for fiber reorientation with stretch, reminiscent of immature valves (**Fig. 3F**). Taken together, these results suggest that valve tissue microstructure is similarly sensitive to changes in deformation even at low strain and high stiffness zones.

### Age Dependent Changes in Anterior Mitral Valve Leaflet Extracellular Matrix Composition in Wild-type C57BL/6J Mice

**Figure 4:**
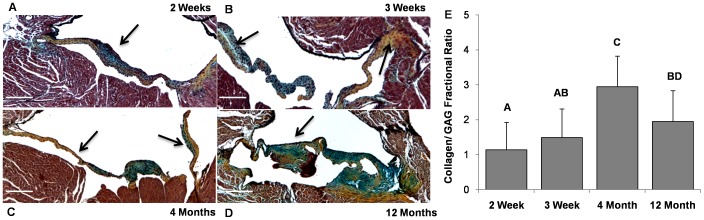
Temporal histological examination of C57/B6J mitral valves. (A) 2 week old murine mitral valves contain similar fractional amounts of collagen and GAGs, with the majority of collagen near the attachment zones but mostly undefined architecture (Movat’s stain: yellow/orange = collagen, green/blue = GAGs). (B) More collagen relative to GAGs is present in 3 week old MV, with matrix stratification developing (Arrows). (C) At 4 months, murine mitral valves have nearly 3 times the amount of collagen to GAGs, are significantly more compact, and have well defined atrialis/fibrosa strata (Arrows). (D) At 12 months, murine mitral valves have significantly less collagen to GAGs, with dramatically increased thickening and reduced structural organization. (E) Digital quantification of matrix composition using color thresholding. Data compared as the ratio of collagen to GAGs within each valve leaflet. Magnification, ×4. Scale bars: 200 µm. Error bars show ±SD, n ≥3 valves per time point. Bars that do not share any letters are significantly different according to a one-way ANOVA with Tukey’s post hoc (p≤0.05).

Noticeable changes in structural organization occurred in mitral valves with age. 2–3 week old mitral leaflets contained equal proportions of collagens and GAGs, with more collagen located near the annular attachment region (**Fig. 4A–B**). At 4 months, valves were significantly more compacted in both the annular attachment and mid-zone regions (**Fig. 4C**). At 10–12 months, valves became thicker, more irregular in cross-section, and a larger disorganization of GAG/collagen architecture near the annulus region (**Fig. 4D**). The fractional ratio of collagen to GAG increased from 2 weeks to 4 months (1.14±0.78 to 2.94±0.88, P<0.05), then tapered off in older valves (1.94±0.89) (**Fig. 4E**). Taken together, these results suggest that mitral valve tissue biomechanics was related to the fractional ratio of collagen to GAGs.

### Increased Compliance of ^C1039G/+^Fbn1 Mitral Valves and its Relationship to Tissue Microstructure

**Figure 5:**
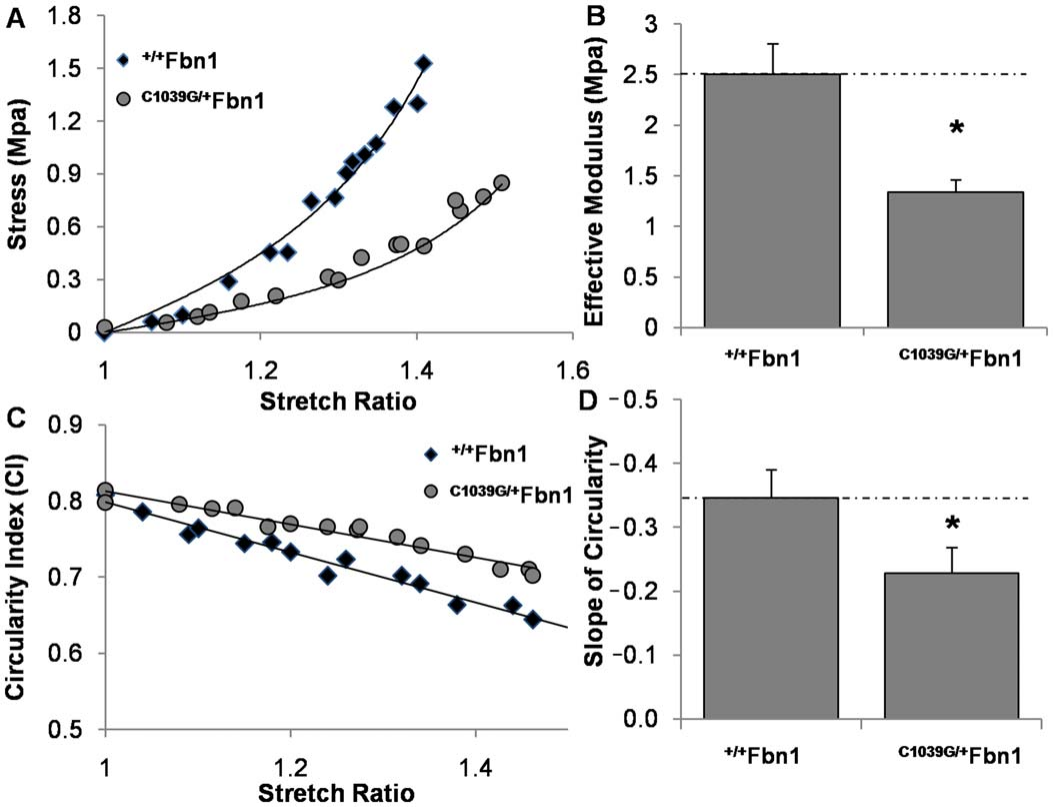
Biomechanical analysis of ^+/+^ *Fbn1* and ^C1039G/+^ *Fbn1* at 4 months. (A) Representative stress-strain responses of mitral valves loaded in the circumferential direction. (B) Stress-strain data were fit to an exponential Fung model, coefficients of which were used to determine effective modulus. (C) Representative stretch induced cell shape changes responses defined by the circularity index (CI) = 4*Pi*(Area/Perimeter^2^). (D) Circularity-index data were modeled as a linear fit, the negative slope of which was used for comparison. Error bars show ±SD, n ≥6 valves per condition. Asterisks signify statistical differences according to a Student’s t-test (p≤0.05).

Transported ^+/+^ *Fbn1* and ^C1039G/+^ *Fbn1* valves had over 90% viability upon arrival, similar to valves excised and assessed without transportation (**Fig. S5**). As anticipated, 4 month old ^+/+^ *Fbn1* valves responded nonlinearly when stretched, with an effective modulus that was not significantly different from those of our locally-housed mouse valves (2.51±0.31 MPa). ^C1039G/+^ *Fbn1* valves, in contrast, had significantly reduced moduli (1.34±0.12 MPa, P<0.05) (**Fig. 5A–B**). At the microstructural level, we found a reduction in the negative slope of circularity index with ^C1039G/+^ *Fbn1* mice relative to ^+/+^ *Fbn1* (–0.23±0.04 vs. –0.34±0.04, P<0.05) (**Fig. 5C–D**), suggesting that the fibrillin-1 mutation impairs local cell sensitivity to tissue stretch. Likewise, fiber alignment with stretch is reduced in ^C1039G/+^ *Fbn1* mitral valves in comparison to wildtype (0.36±0.08 vs. 0.85±0.12, P<0.05) (**Fig. S6**). This suggests that matrix fiber reorganization with stretch is also impaired in mitral valves of *^C1039G/+^Fbn1* mice, which may explain in part their reduced global tissue stiffness.

### Fractional Composition of the Extracellular Matrix Components in the ^C1039G/+^Fbn1 Mitral Valves

**Figure 6:**
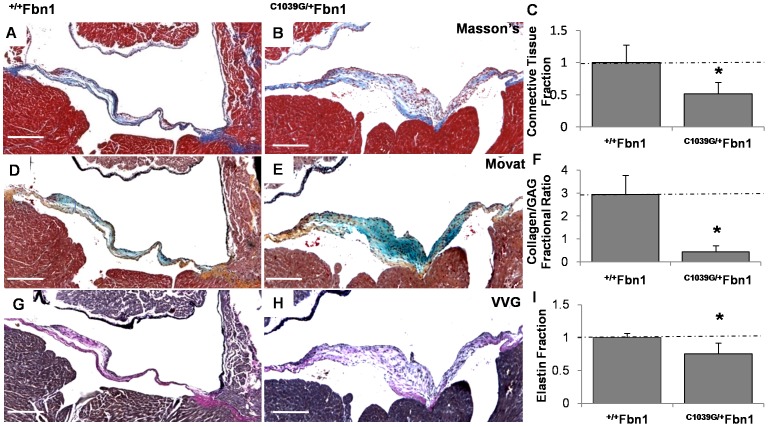
Differences between ^+/+^ *Fbn1* and ^C1039G/+^ *Fbn1* mitral valve matrix composition at 4 months. (A–B) Masson’s Trichrome reveals a reduction in the fractional amount of connective tissue in the marfan mitral valve compared to wildtype. (C) Digital quantification of the connective tissue composition using color thresholding, with blue regions denoting connective tissue. (D–F) Movat’s stain reveals over 3-fold reduction in the fractional amount of collagen compared to GAGs in the Marfan mitral valve relative to the wildtype (yellow-collagen, blue-GAGs). (G–I) Verhoeff’s–van Gieson (VVG) stain reveals a significant reduction in the fractional amount of elastin in the Marfan mitral valve compared to the wildtype (purple/black-elastin). Magnification, ×4. Scale bars: 200 µm. Error bars show ±SD, n ≥3 valves per condition. Asterisks denote statistical differences according to a Student’s t-test (p≤0.05).

The fraction of connective tissue was found significantly less in the *^C1039G/+^Fbn1* (0.51±0.18) compared to the ^+/+^ *Fbn1* leaflets (1±0.28, P<0.05) (**Fig. 6A–C**). Less compacted collagen was present at the attachment zone, but extensive GAGs were present throughout the leaflet with a noticeable reduction in the fibrosa layer. The collagen to GAG fractional ratio was significantly less in the ^C1039G/+^ *Fbn1* (0.44±0.26) compared to the ^+/+^ *Fbn1* (2.94±0.84, P<0.05) (**Fig. 6D–F**). In contrast to the layer specific elastin present in wildtype valves, mutant valves contained significantly less fraction of elastin (0.75±0.17 for C1039G/+ compared to 1±0.07 for wildtype, P<0.05), which was less organized throughout the leaflet (**Fig. 6G–I**). Collectively, these results indicate that decreased stiffness with *Fbn1* mutation correlates with a reduction in the amount of connective tissue fraction, elastin fraction, and collagen/GAG fractional ratio.

## Discussion

Myxomatous degeneration of the MV with consequent valve prolapse and dysfunction is a common phenotype, affecting 2–3% of individuals in the general population [3]. Over the last decade, murine models have been indispensible for understanding many of the functional consequences of mutations in fibrillin-1 [13], [21], [22]. The bulk of these studies have established the critical contribution of fibrillin-1 deficiency to disease progression through altered cell–matrix interactions and dysregulated TGFβ signaling. To further evaluate the global biomechanical and local cell-matrix relationships, we developed a novel method to evaluate mouse MV architecture during loaded conditions. We found that in the *Fbn1* mutant mice, the effective modulus was less, which was supported by our histological analysis revealing a reduction in the amount of connective tissue fraction, elastin fraction, and collagen/GAG fractional ratio. Interestingly, both valve deformation and fiber alignment were significantly reduced, suggesting that MV cell-matrix interactions become progressively more decoupled. Quantitative differences between local cell or matrix deformations and global tissue stretch may be an important contributor to pathological tissue remodeling in valves, whether originated by aging, mechanical, or genetic mutation contexts.

The ability to investigate the integration of mechanics and genetics in small animal models across multiple length scales represents a powerful tool for quantifying functional consequences of sub-lethal mutations. Historically, this has been particularly challenging due to the difficulty with attaching small tissues (0.2 mm–2 mm), measuring force ranges (10 µN–10 mN), and imaging of underlying microarchitecture. Pipette aspiration has been a classic approach for measuring the mechanical properties of small tissues. Butcher et al. used this method to measure the nonlinear pseudoelastic biomechanics of developing chick atrioventricular cushions while, Krishnamurthy et al., used a similar approach to measure the measure regional mechanical properties of mouse aortic valve tissue [20], [23]. Others have used micro-indentation testing systems to determine the compressive and biphasic mechanical properties of cartilage in the small joints of the mouse using glass fibers [24]. More sophisticated devices have been designed to take advantage of the cylindrical geometry of soft tissues, such as blood vessels. Small caliber (50–5000 µm) blood vessels mounted on cannulaes provide a substrate for well-controlled mechanical loads and multiaxial mechanical tests [25]. While these approaches provide tremendous information in small model tissue biomechanics, quantification of the underlying cell deformation is necessary for understanding the mechanobiological consequences of tissues at physiological levels. To overcome these limitations, a novel stretching apparatus and gripping method was implemented to directly quantify both global tissue biomechanics and local cellular deformation and matrix fiber realignment in murine mitral valves.

The clinical manifestation of the MV when it is affected in MFS involves redundant prolongation of one or both leaflets, often accompanied by myxomatous thickening [26]. MV prolapse and regurgitation also commonly occur in related conditions. A retrospective review and ultrasound based comparison of MV morphology at the time of MV surgery in MFS and myxomatous non-syndromic MV disease reported that individuals with MFS presented at an earlier age (41 vs. 57 years, respectively), and that the anterior MV leaflet was longer in MFS than in non-syndromic MV disease (40 vs. 33 mm, respectively) [27]. As with many phenotypic characteristics of MFS, the presence and severity of MV prolapse, thickening, and regurgitation often vary within families segregating the same *Fbn1* gene mutation [28], [29]. In mice, MV leaflets heterozygous or homozygous for a *Fbn1* mutation were also shown to be both longer and thicker than in wild-type littermates, and these differences correlated with increased TGFβ signaling and increased production of collagen. Adult heterozygous mutant mice were also shown to have MV prolapse by high-resolution echocardiography [13]. Furthermore, in sheep with displaced papillary muscles (commonly used to replicate MV regurgitation), assessment of total mitral leaflet area by 3-D echo was found to increase with stretch, and was 2.8 times thicker than normal [30]. While further investigation is needed, these findings suggest that adaptive mechanisms within the MV are connected to the in-vivo physiological loads within the MV. In the case of the *Fbn1* mutation, increased leaflet length/thickness, annulus diameter, and transvalvular pressure all govern the stress distribution within the MV (tissue deformation), which potentially leads to valve remodeling and aberrant function.

Recent studies have investigated both the in-vitro and in-vivo dynamic deformation of the MV. It was found that the MV anterior leaflet experiences large anisotropic strains, approaching stretch ratios of 1.2 in the circumferential and 1.4 in the radial direction at physiological levels [31]. Furthermore, their observations also suggest that changes in left ventricular pressure and annular geometry result in altered effective leaflet stiffness, which may be an important factor in reducing leaflet stress [32]. From our studies, the decreased compliance of *Fbn1* MV implies they may be subjected to increased strain at the same transvalvular pressures. However, we find that the interstitial cells within *Fbn1* valves do not deform to the same degree at the same strain. These two datasets suggest there may exist a balance between excess loading and altered mechanosensitivity that can initiate or exacerbate pathological cell remodeling. If the known differences in growth factor expression, particularly TGFβ, is additionally factored in, clearly uncovering the mechanobiological mechanism of MV dysfunction in MFS remains a major challenge. The additional mechanical tools generated from this study enable important new hypotheses and avenues of research in this direction.

Our histochemical analysis also revealed a large abundance of GAGs, which was not unexpected as myxomatous valves have been shown to contain more GAGs and PGs than normal [8], [33]. In MV excised from human patients, myxomatous leaflets were found to have 3% more water content and 30% higher GAG concentrations than normal mitral leaflets, which are thought to influence the hydration-related “floppy” nature of these tissues [34]. Biomechanical testing of myxomatous valves have also been shown physically and mechanically different from normal MV leaflets in human patients such that they are more extensible and less stiff [35]. These findings align closely with our results, which suggest that the functional consequence of valve thickening, reduced fiber alignment, and abnormal matrix deposition ultimately leads to a reduction in tissue stiffness. Upon substantial remodeling, the functional consequence of MV leaflets can ultimately result in prolapse and regurgitation [36]. Although several studies have reported disparate prevalence of MV prolapse among individuals with MFS, the biomechanical remodeling within the MV is substantial [37]. We suspect the underlying mechanobiological regulation of VIC is a critical component for understanding the long-term biomechanical and pathological consequences of MV disease in MFS.

Interestingly, at the cellular level, we found that both collagen fiber alignment and overall cellular deformation were significantly reduced in the *Fbn1* mutant leaflets. Although the underlying mechanism is currently unknown, cellular deformation has been shown dependent upon integrin binding and traction force generation. Numerous studies have shown that the degree of traction force is proportional to substrate stiffness, via integrins [38]. Strengthening of focal adhesions is thought to dominate on stiffer substrates as compared to soft substrates [39]. Bax et al., reported an RGD sequence of fibrillin-1 that supports cellular adhesion in vitro via integrin αvβ3. Using human dermal fibroblasts, fibrillin-1 protein fragments induced signaling events that led to cell spreading, altered cytoskeletal organization, and enhanced extracellular fibrillin-1 deposition [40]. Mutations in integrin binding also result in a unique human phenotype called “stiff skin syndrome (SSS)”. Cultured dermal fibroblasts from patients with SSS showed dysregulated amounts activated (phosphorylated) focal adhesion kinase (pFAK) [41]. Therefore, the increased GAG content, disorganized extracellular matrix, and/or altered integrin motifs may regulate traction force generation, and hence the ability to deform during mechanical load.

Few studies have investigated the mechano-regulation of VIC in response to altered pressure or strain. VIC isolated in vitro and mechanically strained at 10%, 14%, and 20% have also been shown to upregulate collagen synthesis by an increase in [3H]-proline incorporation in a strain dependent manner [42]. Hence, collagen synthesis by VICs is dependent upon the degree and duration of stretch. Furthermore, when pulmonary VICs were exposed to aortic pressure levels, as occurs following the Ross operation, collagen and sulfated glycosaminoglycan synthesis were increased significantly [43]. This demonstrates that VICs are capable of remodeling the ECM in response to changes in strain magnitude and/or the stress state of the tissue. Comparing our findings between age and *Fbn1* mutation, we find a strong similarity between altered local cell-matrix kinematics and changes in matrix composition. Interestingly, leaflet thickness, blood pressure, and MV annulus were also correlated [15], [44], [45]. This suggests that elevated blood pressure magnitudes (age dependent) and/or altered valve stress states may dictate the mechano-biological response, possibly through regulating ECM composition and hence, overall cellular deformation.

Taken together, an important question previously unidentified and revealed by this study is whether these alterations in local cell-matrix interactions with stretch are a cause or consequence of valve tissue remodeling. Three possible hypotheses into the mechanical etiologies of MFS valve dysfunction include 1) the interstitial cells experience a reduced mechanical force distribution in *Fbn1* mutant vs. wild-type tissue, 2) cells in both cases experience the same loads but, the *Fbn1* mutant interstitial cell is unable to sense it correctly (e.g. its mechanotransduction processes are tuned improperly), or 3) both cells sense the same loads but the *Fbn1* mutant cells can’t respond appropriately, pathologically remodeling the matrix instead of augmenting/maintaining its mature organization. Studies have shown that cytokines and growth factor signaling is altered in *Fbn1* mutant valves, but it is yet unclear whether they are genetically prescribed or the consequence of the altered mechanobiological signaling (i.e. integrin binding or expression). Further investigation systematically testing these different hypotheses is ongoing.

Our results provide a framework to explore a fibrillin-1 mutation leading to abnormal biomechanics/mechanobiology and clinical manifestations in MFS associated MV disease. The most novel and unexpected finding is that not only are extracellular matrix components altered, but the response of VIC to stress is also changed, so that cells effectively sense less stretch in MFS during normal physiological loads. This decoupling of local cell and matrix kinematics from global valve tissue stretch may have direct implications in the ability of VIC to maintain normal homeostatic cellular deformation. Pathological changes in MV matrix composition and organization ensue, which in turn weakens the tissue. These results support the potential for matrix stabilization techniques such as collagen crosslinking or radiofrequency ablation for maintaining mechanical performance and cell mechanobiology, and thereby delay pathological remodeling [46].

### Study Limitations

The mouse MV is an extremely thin piece of tissue, with an average thickness in the mid region of ≈30–40 µm (4–5 cells thick), compared to the ≈500–700 µm thick human MV [47]. We therefore used confocal z-stacks to capture both collagen fibers and cells across the entire thickness. With this method, we found that collagen fiber alignment and un-crimping occurred simultaneously across the valve (**Fig. 2E–L**), but potential thickness dependent inhomogeneities were not addressable. We also measured the circularity index (CI) of cells rather than nuclear aspect ratios (NAR). While CI is representative of cell morphology, cells were well spread in almost all cases, potentially limiting our sensitivity to deformation. However, using the change in CI, we were able to ascertain significant differences in local cell behavior in association with both age and *Fbn1* genotype.

### Conclusion

Degenerative biomechanical remodeling of the MV is a key component of Marfan syndrome. Through quantifying global biomechanical and local cell-matrix relationships in *Fbn1* mutant murine mitral valves, we found that local cell and matrix kinematics are decoupled to different degrees correlating with magnitude of global valve tissue stretch. Changes in local cell-matrix deformation relationships may be an important metric for determining mechanisms of normal and pathological tissue remodeling in valves, as in aging or genetic mutation.

## Supporting Information

Figure S1 **Silicon post construction and calibration.** (A) Elastomeric posts mounted on micro-manipulation and deflection force measured via weight scale. (B) Representative calibration curves for fixed aspect ratio of height to diameter (H/D) = 3 with varying diameters. Euler beam theory (dashed line) predicts a linear increase in bending stiffness with diameter. Post bending stiffness was measured for 3 diameters and agrees with theory. n = 6 (3 silicon batches), with data presented as mean± SD.
(TIF)

Figure S2 **Stretching mouse valves under continuous fluorescence imaging.** (A) Post deflection used for measuring force generation. (B) Top view of post with attached tissue (center-outlined). Outline of post cross-section included as a reference (circles). (C) Macro scale valve deformation at 10X under confocal microscropy (extracellular matrix-green, cells- red). Stars denote post centroids. (D) Valve thickness measured using confocal microscopy full thickness z-stacks (40 um in this image).
(TIF)

Figure S3 **Digital quantification of ECM composition.** Serial sections of the mitral valves were stained with Movat’s Pentachrome to identify the relative amounts of cells, collagen, and glycosaminoglycans at each age. The relative contributions changed dramatically across the length of the valve, so the entire valve area was considered (dashed contour). Colors were separated using an RGB or CMYK channel splitter. Black and white thresholds were created in NIH ImageJ and used to determine the volume fractions of each contributor.
(TIF)

Figure S4 **Biomechanical analysis of 10 month C57BL/6J mitral valves.** (A) Representative stress-strain curves of mitral valve loaded in the circumferential direction. (B) Representative circularity index curve as defined by the ratio of area to perimeter squared. (C) Representative fiber-alignment curve as defined by the (D) Fourier-Transform and presented as histograms.
(TIF)

Figure S5 **Live/dead stain on shipped ^C1039G/+^** ***Fbn1*** **mitral valves.** (A) Visualization of Live/dead stain. (B) Shipped FBN1 valves had over 90% viability upon arrival, similar to valves excised directly at our institution. Magnification, ×4 Scale bars: 500 µm. Error bars show ±SD, n ≥3 valves per time point.
(TIF)

Figure S6 **Fiber alignment analysis of ^+/+^** ***Fbn1*** **and ^C1039G/+^** ***Fbn1*** **at 4 months.** (A) Representative fiber-alignment curve as defined by the Fourier-Transform. (B) Fiber-alignment curves were modeled as a linear fit and the negative slope was used for comparison. Error bars show ±SD, n ≥6. Asterisks signify statistical differences according to a Student’s t-test (p≤0.05).
(TIF)

Materials S1 **Extended methods describing mouse valve viability, testing, and device fabrication.**
(DOC)
