# Supplementary figures and images for: Multi-Scale Biomechanical Remodeling in Aging and Genetic Mutant Murine Mitral Valve Leaflets: Insights into Marfan Syndrome
Source: PLoS One. 2012 Sep 11;7(9):e44639. doi: 10.1371/journal.pone.0044639 (PMC3439411; doi:10.1371/journal.pone.0044639)

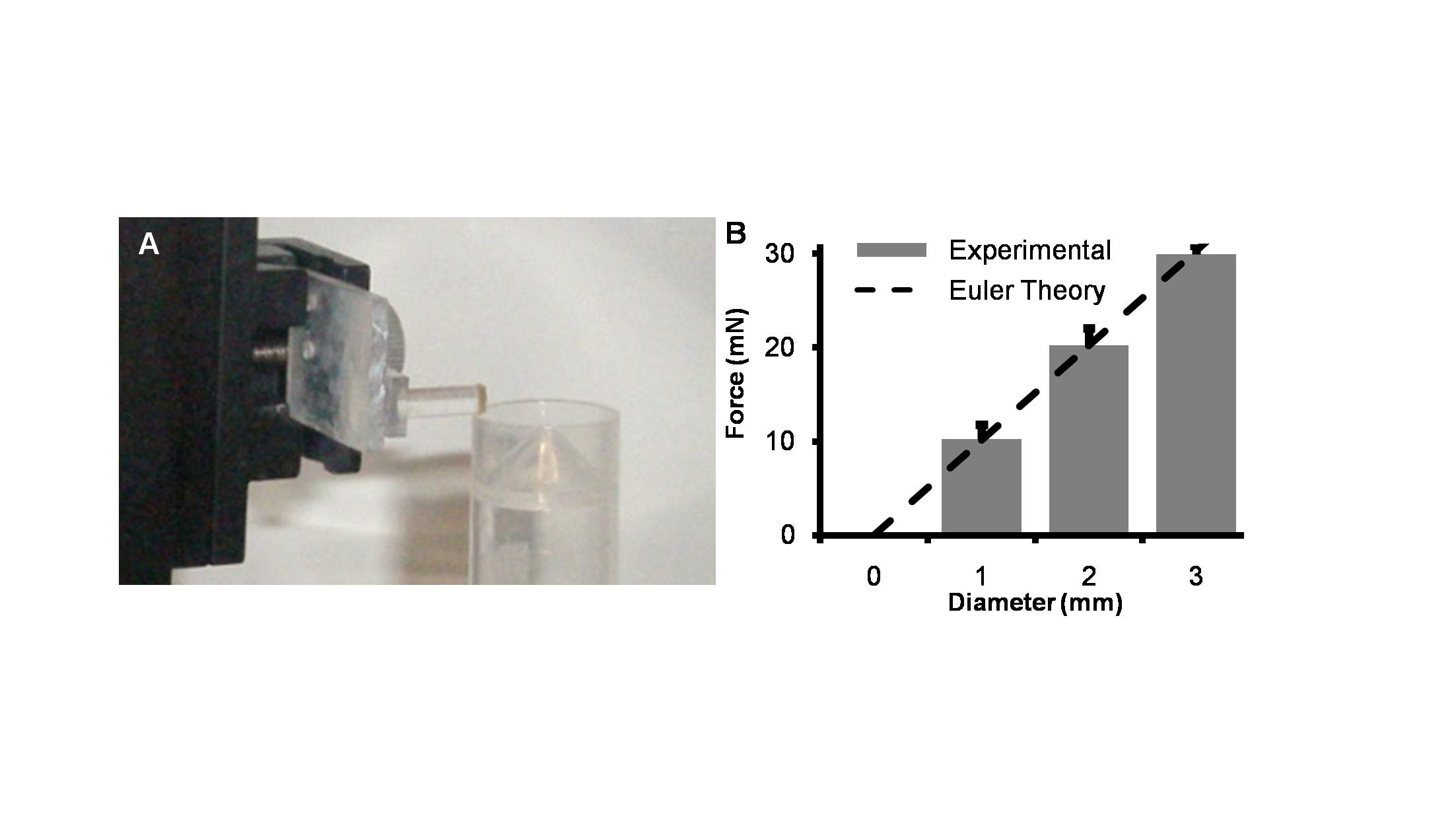

Supplement: Figure S1 — Silicon post construction and calibration. (A) Elastomeric posts mounted on micro-manipulation and deflection force measured via weight scale. (B) Representative calibration curves for fixed aspect ratio of height to diameter (H/D) = 3 with varying diameters. Euler beam theory (dashed line) predicts a linear increase in bending stiffness with diameter. Post bending stiffness was measured for 3 diameters and agrees with theory. n = 6 (3 silicon batches), with data presented as mean± SD. (TIF) [file pone.0044639.s001.tif]

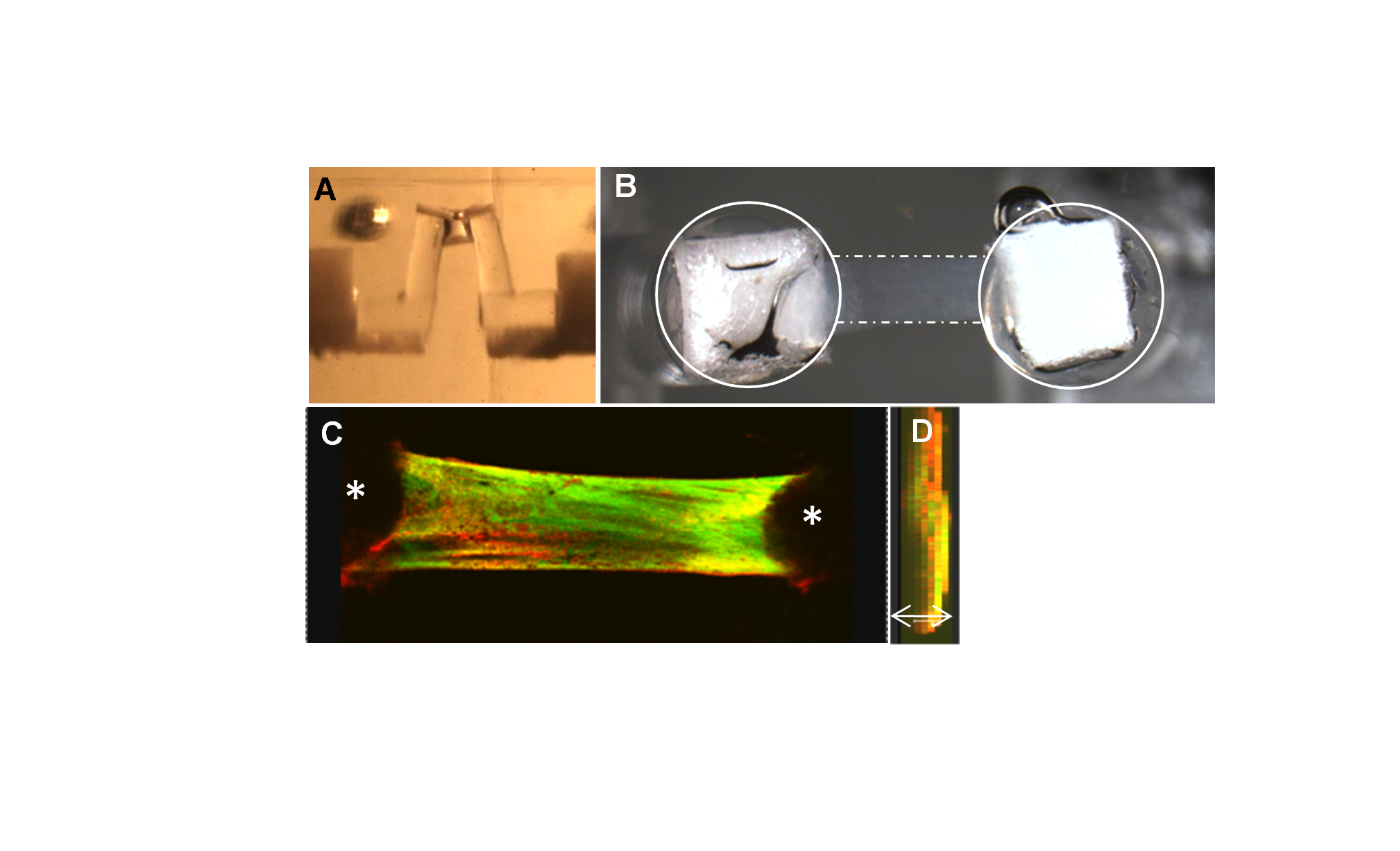

Supplement: Figure S2 — Stretching mouse valves under continuous fluorescence imaging. (A) Post deflection used for measuring force generation. (B) Top view of post with attached tissue (center-outlined). Outline of post cross-section included as a reference (circles). (C) Macro scale valve deformation at 10X under confocal microscropy (extracellular matrix-green, cells- red). Stars denote post centroids. (D) Valve thickness measured using confocal microscopy full thickness z-stacks (40 um in this image). (TIF) [file pone.0044639.s002.tif]

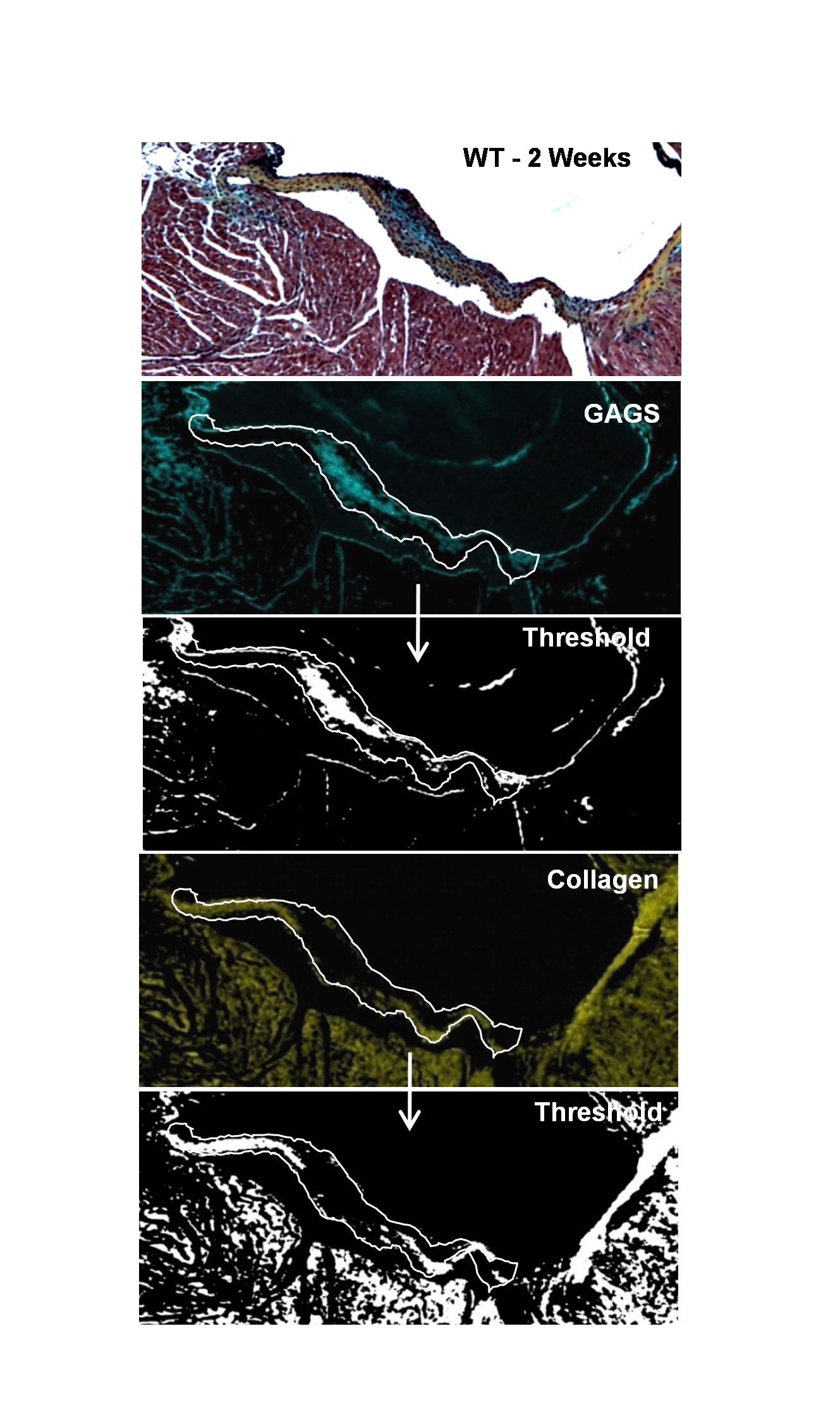

Supplement: Figure S3 — Digital quantification of ECM composition. Serial sections of the mitral valves were stained with Movat’s Pentachrome to identify the relative amounts of cells, collagen, and glycosaminoglycans at each age. The relative contributions changed dramatically across the length of the valve, so the entire valve area was considered (dashed contour). Colors were separated using an RGB or CMYK channel splitter. Black and white thresholds were created in NIH ImageJ and used to determine the volume fractions of each contributor. (TIF) [file pone.0044639.s003.tif]

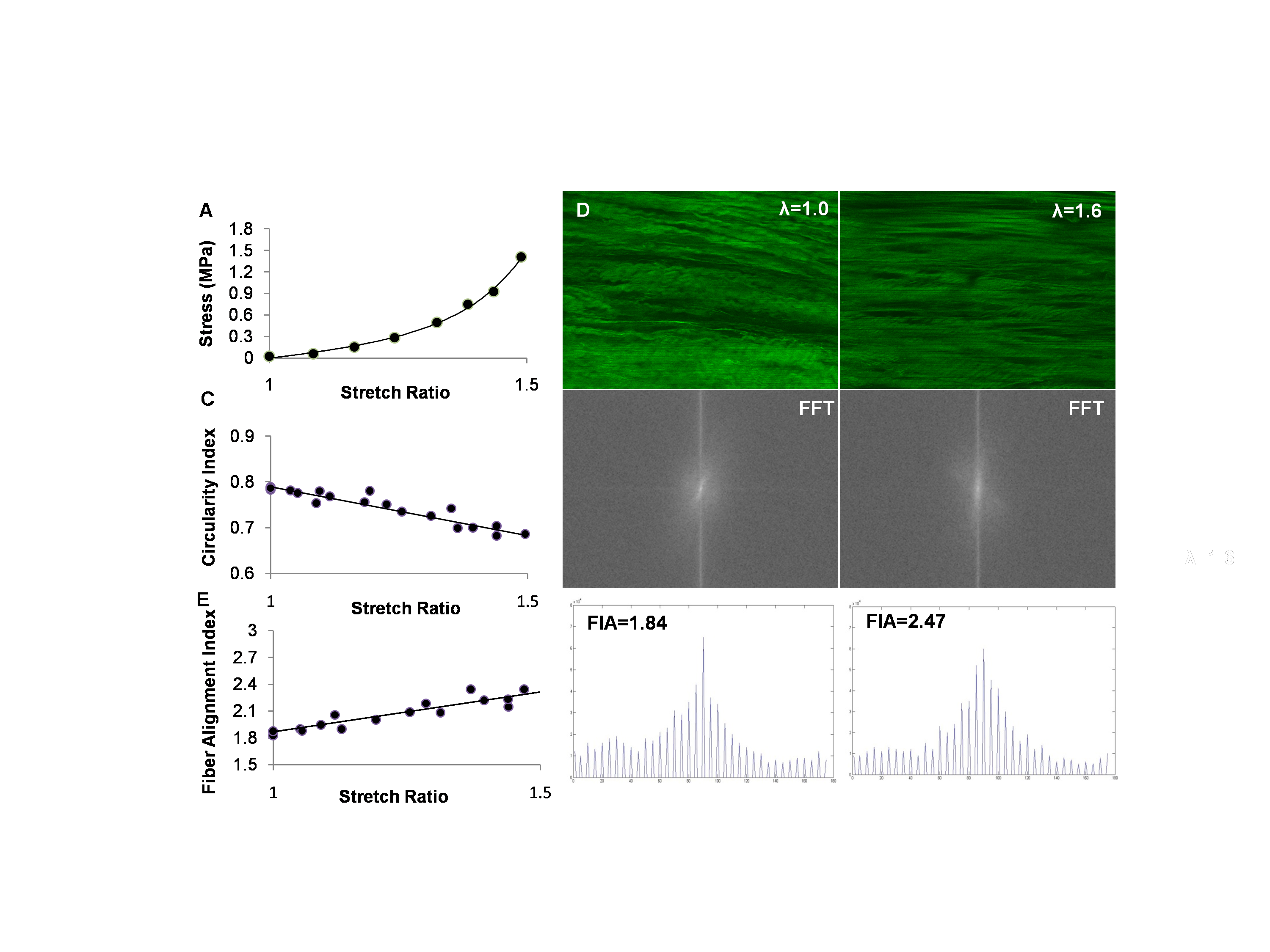

Supplement: Figure S4 — Biomechanical analysis of 10 month C57BL/6J mitral valves. (A) Representative stress-strain curves of mitral valve loaded in the circumferential direction. (B) Representative circularity index curve as defined by the ratio of area to perimeter squared. (C) Representative fiber-alignment curve as defined by the (D) Fourier-Transform and presented as histograms. (TIF) [file pone.0044639.s004.tif]

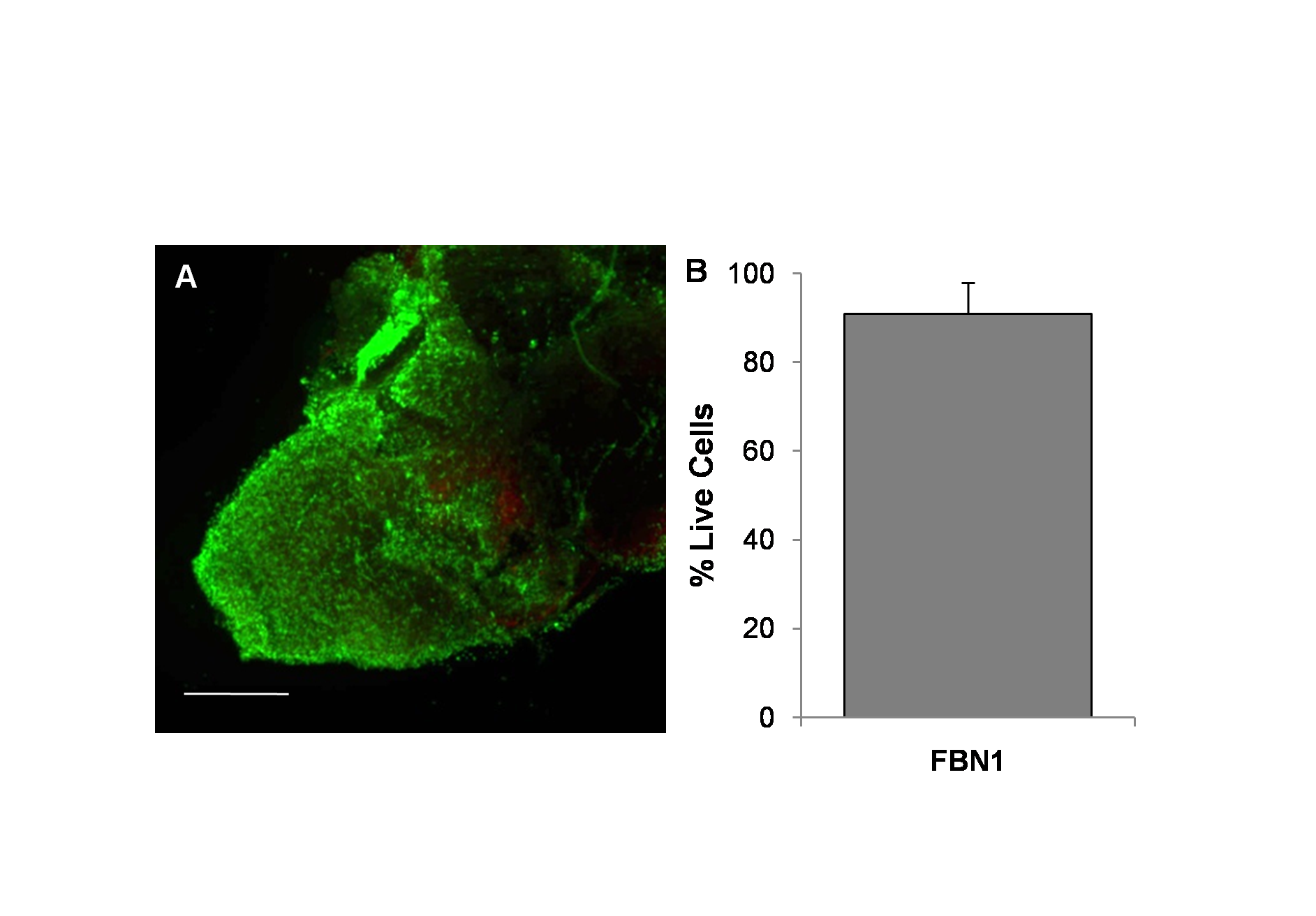

Supplement: Figure S5 — Live/dead stain on shipped C1039G/+ Fbn1 mitral valves. (A) Visualization of Live/dead stain. (B) Shipped FBN1 valves had over 90% viability upon arrival, similar to valves excised directly at our institution. Magnification, ×4 Scale bars: 500 µm. Error bars show ±SD, n ≥3 valves per time point. (TIF) [file pone.0044639.s005.tif]

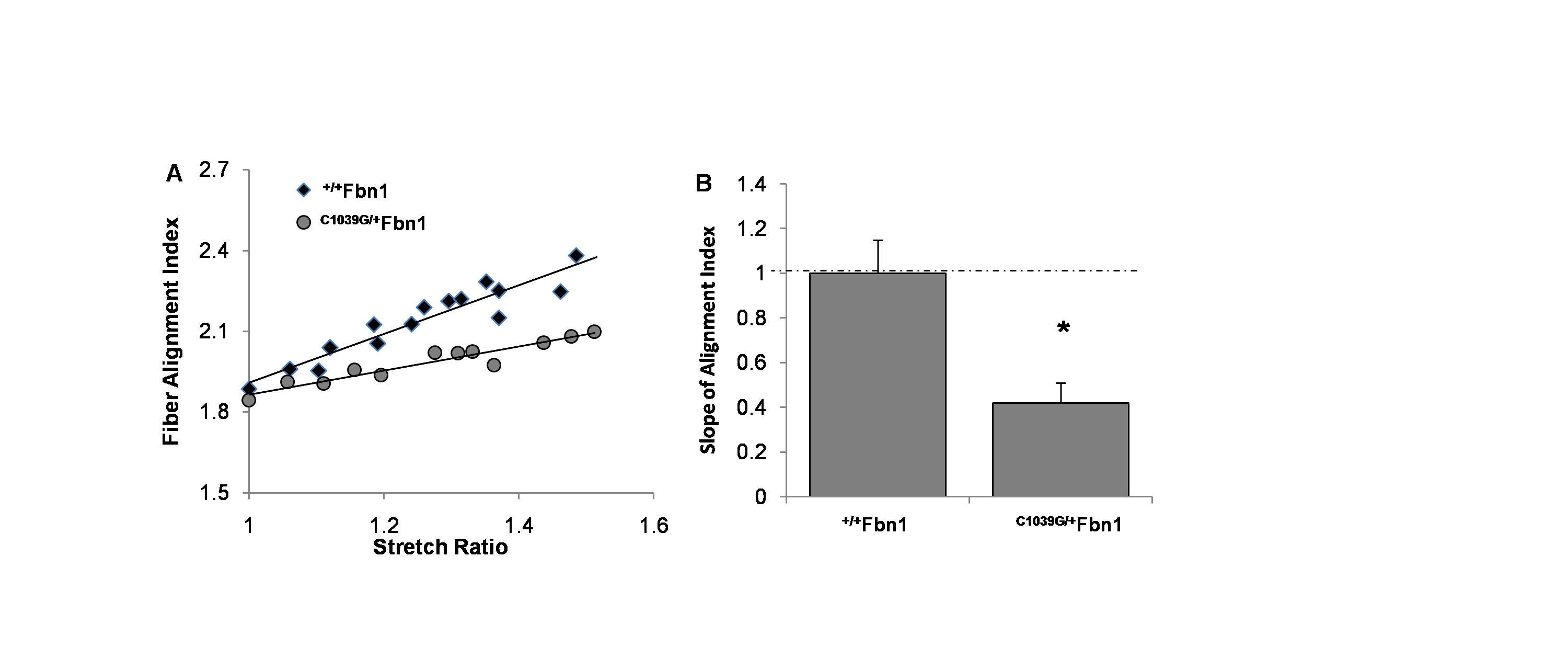

Supplement: Figure S6 — Fiber alignment analysis of +/+ Fbn1 and C1039G/+ Fbn1 at 4 months. (A) Representative fiber-alignment curve as defined by the Fourier-Transform. (B) Fiber-alignment curves were modeled as a linear fit and the negative slope was used for comparison. Error bars show ±SD, n ≥6. Asterisks signify statistical differences according to a Student’s t-test (p≤0.05). (TIF) [file pone.0044639.s006.tif]
