# Supplementary material for: Multi-Scale Biomechanical Remodeling in Aging and Genetic Mutant Murine Mitral Valve Leaflets: Insights into Marfan Syndrome
Source: PLoS One. 2012 Sep 11;7(9):e44639. doi: 10.1371/journal.pone.0044639 (PMC3439411; doi:10.1371/journal.pone.0044639)
Supplement: Materials S1 — Extended methods describing mouse valve viability, testing, and device fabrication. (DOC) [file pone.0044639.s007.doc]

**SUPPLEMENTARY MATERIAL**

**Mitral Valve Viability**

Wildtype (FBN1 +/+) and heterozygous (FBN1 C1039G/+) knock-in mice between the ages of 2 weeks and 10 months were used in this study, as that reflects a broad range of murine valve growth and development^1^. Details of the human *Fbn1* mutation knock-in (C1039G/+) have been previously described^2^. Mutant mice were back-crossed into the C57BL/6J (Jackson Laboratory, 000664) background for at least 10 generations. All animal use was approved by the Cornell University and Johns Hopkins Institutional Animal Care and Use Committee (IACUC). For valve tissue isolation, mice were then anesthetized using anintraperitoneal injection of sodium pentobarbital (125 mg/kg) and verified via toe pinch. Acranio-caudal incision was then made to expose the thoracic cavity, and the diaphragm severed. The heart was then excised above the level of the aortic valve and perfused with ice-cold saline to wash away any blood from the chamber and valve regions. Mutant mouse hearts were prepared at Johns Hopkins, placed in a 15ml conical tube containing DMEM, 10% FBS, and 0.1% ITS, and shipped on ice the same day to Cornell. Shipped mitral valve tissues were stained with Live-Dead to confirm greater than 90% cell viability. Tissues were tested immediately on arrival, which was within 24 hours after the initial isolation.

**Device and Silicone Post Fabrication.** We designed and fabricated a device that miniaturized a classical uniaxial strip test (Fig. 1A). Two challenges for very small, ultra compliant tissues are 1) measuring very small magnitude forces applied, and 2) securing the tissue to a member that can stretch without failure at the grips. We addressed the first using classical Euler cantilever beam deflection mechanics. Elastomeric posts (Polydimethylsiloxane, Sylgard184) were prepared by casting a 10:1 mix of base and curing agent in custom made negative molds and curing at 65^o^C for 6 hours. The mold geometry (cross-section, depth) was adjusted to create posts with different force sensitivity and strain capacity. Bending stiffness was calculated analytically with the measured deflection (Fig. 1B), and measured force verified independently via mass balance (Fig. S1) as previously described ^3^. Cylindrical posts with a diameter of 2mm and axial length of 6mm were used for mitral valve testing. Once calibrated, the silicon posts were mounted on top of two L-shaped plastic beams that traveled on linear rail guides. A screw-driven wedge enabled equal and opposite co-linear displacement of the L-beams, and thus the cantilever posts, micron resolution (Fig. 1C-D). Valve tissue was secured to the post top using filter paper and a high-viscosity cyanoacrylate (454 Prism, Loctite). The tissue was kept flat prior to mounting by first placing it on a glass coverslip and removing all excess liquid, then inverting it to contact the posts. Once mounted, the entire system was placed on the stage of an upright confocal microscope (TCS SP2, Leica).

**Mitral Valve Isolation and Biomechanical Testing.** Wildtype (*Fbn1* +/+) and heterozygous ( ^C1039G/+^*Fbn1*) knock-in mice between the ages of 2 weeks and 10 months were used in this study, as that reflects a broad range of murine valve growth and development ^1^. Details of the human *Fbn1* knock-in mutation (C1039G/+) have been previously described ^2^. The anterior mitral valve leaflet was dissected from the annular wall and trimmed to a rectangular strip using micro-scissors. The presence and architecture of cells and extracellular matrix were visualized using fluorescent vital dyes (5 µM CellTrackerRed CMPTX and 10 µM 5-DTAF, respectively, both from Invitrogen). Leaflets were then mounted to the posts in the atrialis-up configuration, immersed in a bath containing Dulbecco’s phosphate buffered saline (DPBS) and placed under an upright confocal (TCS SP2, Leica). Zero-strain reference length was defined as the maximum length of tissue for which no post deflection was visible (representing a maximum tensile preload of 100 nN). Tissues were first preconditioned with 3 cycles of approximately 10% strain, followed by a single quasi-static tensile test administered at 25-micron displacement intervals. At each test position, a macroscopic tissue view of the valve was taken at 10x magnification, and microscopic z-stacks of cells and fibers at 40x, both using confocal microscopy. Simultaneously, post deflection was monitored using a digital SLR camera (D5000, Nikon) positioned at the side of the test system. All mouse work was conducted with Institutional Care and Use Committees (IACUC) approval.

**Determination of Local Biomechanics**. Biomechanical parameters were quantified through image analysis performed using ImageJ (NIH). Global tissue deformation was measured via tip-to tip post displacement, while local tissue deformation at each incremental stretch were measured at the midline zone by tracking virtual fiducial markers using confocal microscopy as previously described ^4^. Applied force was determined by comparing measured post-deflection against the calibration curve. Tissue width and thickness was measured at the initial state using confocal microscopy (10X and 40x z-stacks, respectively). First Piola-Kirchoff stress and stretch ratio-based strain data were generated and fit to a uniaxial exponential Fung model. From this, an effective tissue modulus was determined using the two material coefficients (α,β) as previously described ^5^. Local matrix fiber alignment and cell deformation was captured at each stretch interval using 40x confocal z-stacks (Fig. S2). Cell shape changes were traced manually, tracked over subsequent images, and quantified as a cell circularity index (CI = 4*Pi*(Area/Perimeter^2^)) as manually traced in ImageJ. Fiber alignment along the axis of stretch was quantified as an alignment index (FAI) custom algorithm through MATLAB as previously described ^6^. The Fourier Transform intensity was computed between 0^o^ to 180^o^ (angles with respect to horizontal) at 5^o^ intervals and presented the results in the form of a histogram as a graphic display of alignment along a particular angle. The AI integrals were approximated as the sum of these 5^o^ intervals. This was directly compared to the summed angles ±10 ^o^ from the direction of stretch for a FAI.

**Extracellular Matrix Composition.** Additional hearts were excised and fixed in 10% buffered formalin overnight, arranged in 1.5% agar prior to paraffin embedding, and slides were prepared using 7µm sections. Sections of paraffin-embedded tissue were prepared and stained with either Movat Pentachrome, Masson’s Trichrome, or Verhoff-van Gieson stain (VVG). Mitral valves were examined at 4X magnification and compared to a 200µm calibration standard included in each image. Only sections in which the entire anterior leaflet was present were included in analyses. Colors representing different matrix constituents were separated in NIH ImageJ using an RGB or CMYK channel splitter and converted to grayscale images. These areas were then thresholded, quantified, and normalized against the area of the entire valve leaflet to determine relative fractional composition as previously described ^7^ (Fig. S3).

**REFERENCES**

1 Hinton, R. B., Jr. *et al.* Mouse heart valve structure and function: echocardiographic and morphometric analyses from the fetus through the aged adult. *Am J Physiol Heart Circ Physiol* **294**, H2480-2488, doi:91431.2007 [pii]

10.1152/ajpheart.91431.2007 (2008).

2 Judge, D. P. *et al.* Evidence for a critical contribution of haploinsufficiency in the complex pathogenesis of Marfan syndrome. *J Clin Invest* **114**, 172-181, doi:10.1172/JCI20641 (2004).

3 Sasoglu, F. M., Bohl, A. J. & Layton, B. E. Design and microfabrication of a high-aspect-ratio PDMS microbeam array for parallel nanonewton force measurement and protein printing. *J Micromech Microeng* **17**, 623-632, doi:10.1088/0960-1317/17/3/027 (2007).

4 Helmke, B. P., Goldman, R. D. & Davies, P. F. Rapid displacement of vimentin intermediate filaments in living endothelial cells exposed to flow. *Circ Res* **86**, 745-752 (2000).

5 Fung, Y. C. Elasticity of soft tissues in simple elongation. *Am J Physiol* **213**, 1532-1544 (1967).

6 Bowles, R. D., Williams, R. M., Zipfel, W. R. & Bonassar, L. J. Self-assembly of aligned tissue-engineered annulus fibrosus and intervertebral disc composite via collagen gel contraction. *Tissue Eng Part A* **16**, 1339-1348, doi:10.1089/ten.TEA.2009.0442 (2010).

7 Butcher, J. T., McQuinn, T. C., Sedmera, D., Turner, D. & Markwald, R. R. Transitions in early embryonic atrioventricular valvular function correspond with changes in cushion biomechanics that are predictable by tissue composition. *Circ Res* **100**, 1503-1511, doi:CIRCRESAHA.107.148684 [pii]

10.1161/CIRCRESAHA.107.148684 (2007).
